# Basic and clinical immunology – 3030. Subcutaneous immunoglobulin therapy: An option for patients who have experienced thrombotic complications with intravenous therapy

**DOI:** 10.1186/1939-4551-6-S1-P205

**Published:** 2013-04-23

**Authors:** Leslie Anne Moss, Paul Keith

**Affiliations:** 1 Allergy and Clinical Immunology, Mcmaster University, Hamilton, ON, Canada

## Background

We report the effective and safe use of subcutaneous immunoglobulin (SCIG) therapy in a patient who suffered stroke following replacement intravenous immunoglobulin (IVIG) infusions for common variable immune deficiency (CVID) at age 28 years.

## Methods

CVID is one of the 6 FDA-approved uses for IVIG, and is required lifelong in these patients. Systemic adverse reactions occur in approximately 2-6% of infusions ^1^, while thrombotic events are very rare. A Therapeutic dilemma arose when our patient had a stroke after an IVIG infusion.

After the stroke, our patient’s IgG level fell to 1.17 g/L, resulting in recurrent pneumonia. Cautious doses of IVIG were restarted and the patient remained on warfarin. These were tolerated well for 13 years. With the introduction of higher concentrations of IgG available for subcutaneous administration, we transitioned the patient to SCIG since it offered many advantages. The preparation was tolerated well by the patient, resulted in therapeutic trough levels, and was effective at preventing infection. It is also associated with a lower risk of thrombotic complications including stroke theoretically since this is extremely rare, related to the even physiologic nature of serum IG levels, without marked increases in levels following the administration of the SCIG preparation ^2^.

## Results

The patient was cautiously continued on IVIG therapy 7 months after the stroke, with modest doses initially of 20g in 250mL solution q4 weeks (goal trough IgG 5-7 g/L). This dose was effective and tolerated well and it was gradually titrated to higher trough levels 9 years later. With known and theoretical advantages of SCIG therapy, the patient achieved therapeutic trough IG level of 9.5 g/L, and did not have any further thrombotic events or other systemic reactions. The patient continues on q3-4-weekly SCIG infusions that are both safe and effective. This therapy may also offer significant Quality of life (QOL) advantages to the patients and their families.

## Conclusions

We report a CVID patient who had a cerebrovascular accident following an IVIG infusion. He was successfully restarted on IVIG therapy while on warfarin and then switched to SCIG, which he has tolerated well.

